# Supplementary material for: Getting More Out of Biomedical Documents with GATE's Full Lifecycle Open Source Text Analytics
Source: PLoS Comput Biol. 2013 Feb 7;9(2):e1002854. doi: 10.1371/journal.pcbi.1002854 (PMC3567135; doi:10.1371/journal.pcbi.1002854)
Supplement: Dataset S1 — GATE software. Dataset S1 bundled with this paper contains a distribution of GATE (or see http://gate.ac.uk/download/). (TGZ) [file pcbi.1002854.s001.tgz › plos-gate/src/gate/resources/img/crystal-clear-icons-license.html]

Everaldo: Crystal


License

The Crystal Project are released under LGPL.

GNU General Public License.

0. This License Agreement applies to any software library or other program which contains a notice placed by the copyright holder or other authorized party saying it may be distributed under the terms of this Lesser General Public License (also called "this License"). Each licensee is addressed as "you".

   A "library" means a collection of software functions and/or data prepared so as to be conveniently linked with application programs (which use some of those functions and data) to form executables.

   The "Library", below, refers to any such software library or work which has been distributed under these terms. A "work based on the Library" means either the Library or any derivative work under copyright law: that is to say, a work containing the Library or a portion of it, either verbatim or with modifications and/or translated straightforwardly into another language. (Hereinafter, translation is included without limitation in the term "modification".)

   "Source code" for a work means the preferred form of the work for making modifications to it. For a library, complete source code means all the source code for all modules it contains, plus any associated interface definition files, plus the scripts used to control compilation and installation of the library.

   Activities other than copying, distribution and modification are not covered by this License; they are outside its scope. The act of running a program using the Library is not restricted, and output from such a program is covered only if its contents constitute a work based on the Library (independent of the use of the Library in a tool for writing it). Whether that is true depends on what the Library does and what the program that uses the Library does.
1. You may copy and distribute verbatim copies of the Library's complete source code as you receive it, in any medium, provided that you conspicuously and appropriately publish on each copy an appropriate copyright notice and disclaimer of warranty; keep intact all the notices that refer to this License and to the absence of any warranty; and distribute a copy of this License along with the Library.

   You may charge a fee for the physical act of transferring a copy, and you may at your option offer warranty protection in exchange for a fee.
2. You may modify your copy or copies of the Library or any portion of it, thus forming a work based on the Library, and copy and distribute such modifications or work under the terms of Section 1 above, provided that you also meet all of these conditions:

   1. The modified work must itself be a software library.
   2. You must cause the files modified to carry prominent notices stating that you changed the files and the date of any change.
   3. You must cause the whole of the work to be licensed at no charge to all third parties under the terms of this License.
   4. If a facility in the modified Library refers to a function or a table of data to be supplied by an application program that uses the facility, other than as an argument passed when the facility is invoked, then you must make a good faith effort to ensure that, in the event an application does not supply such function or table, the facility still operates, and performs whatever part of its purpose remains meaningful.

   (For example, a function in a library to compute square roots has a purpose that is entirely well-defined independent of the application. Therefore, Subsection 2d requires that any application-supplied function or table used by this function must be optional: if the application does not supply it, the square root function must still compute square roots.)

   These requirements apply to the modified work as a whole. If identifiable sections of that work are not derived from the Library, and can be reasonably considered independent and separate works in themselves, then this License, and its terms, do not apply to those sections when you distribute them as separate works. But when you distribute the same sections as part of a whole which is a work based on the Library, the distribution of the whole must be on the terms of this License, whose permissions for other licensees extend to the entire whole, and thus to each and every part regardless of who wrote it.

   Thus, it is not the intent of this section to claim rights or contest your rights to work written entirely by you; rather, the intent is to exercise the right to control the distribution of derivative or collective works based on the Library.

   In addition, mere aggregation of another work not based on the Library with the Library (or with a work based on the Library) on a volume of a storage or distribution medium does not bring the other work under the scope of this License.
3. You may opt to apply the terms of the ordinary GNU General Public License instead of this License to a given copy of the Library. To do this, you must alter all the notices that refer to this License, so that they refer to the ordinary GNU General Public License, version 2, instead of to this License. (If a newer version than version 2 of the ordinary GNU General Public License has appeared, then you can specify that version instead if you wish.) Do not make any other change in these notices.

   Once this change is made in a given copy, it is irreversible for that copy, so the ordinary GNU General Public License applies to all subsequent copies and derivative works made from that copy.

   This option is useful when you wish to copy part of the code of the Library into a program that is not a library.
4. You may copy and distribute the Library (or a portion or derivative of it, under Section 2) in object code or executable form under the terms of Sections 1 and 2 above provided that you accompany it with the complete corresponding machine-readable source code, which must be distributed under the terms of Sections 1 and 2 above on a medium customarily used for software interchange.

   If distribution of object code is made by offering access to copy from a designated place, then offering equivalent access to copy the source code from the same place satisfies the requirement to distribute the source code, even though third parties are not compelled to copy the source along with the object code.
5. A program that contains no derivative of any portion of the Library, but is designed to work with the Library by being compiled or linked with it, is called a "work that uses the Library". Such a work, in isolation, is not a derivative work of the Library, and therefore falls outside the scope of this License.

   However, linking a "work that uses the Library" with the Library creates an executable that is a derivative of the Library (because it contains portions of the Library), rather than a "work that uses the library". The executable is therefore covered by this License. Section 6 states terms for distribution of such executables.

   When a "work that uses the Library" uses material from a header file that is part of the Library, the object code for the work may be a derivative work of the Library even though the source code is not. Whether this is true is especially significant if the work can be linked without the Library, or if the work is itself a library. The threshold for this to be true is not precisely defined by law.

   If such an object file uses only numerical parameters, data structure layouts and accessors, and small macros and small inline functions (ten lines or less in length), then the use of the object file is unrestricted, regardless of whether it is legally a derivative work. (Executables containing this object code plus portions of the Library will still fall under Section 6.)

   Otherwise, if the work is a derivative of the Library, you may distribute the object code for the work under the terms of Section 6. Any executables containing that work also fall under Section 6, whether or not they are linked directly with the Library itself.
6. As an exception to the Sections above, you may also combine or link a "work that uses the Library" with the Library to produce a work containing portions of the Library, and distribute that work under terms of your choice, provided that the terms permit modification of the work for the customer's own use and reverse engineering for debugging such modifications.

   You must give prominent notice with each copy of the work that the Library is used in it and that the Library and its use are covered by this License. You must supply a copy of this License. If the work during execution displays copyright notices, you must include the copyright notice for the Library among them, as well as a reference directing the user to the copy of this License. Also, you must do one of these things:

   1. Accompany the work with the complete corresponding machine-readable source code for the Library including whatever changes were used in the work (which must be distributed under Sections 1 and 2 above); and, if the work is an executable linked with the Library, with the complete machine-readable "work that uses the Library", as object code and/or source code, so that the user can modify the Library and then relink to produce a modified executable containing the modified Library. (It is understood that the user who changes the contents of definitions files in the Library will not necessarily be able to recompile the application to use the modified definitions.)
      .
   2. Use a suitable shared library mechanism for linking with the Library. A suitable mechanism is one that (1) uses at run time a copy of the library already present on the user's computer system, rather than copying library functions into the executable, and (2) will operate properly with a modified version of the library, if the user installs one, as long as the modified version is interface-compatible with the version that the work was made with.
   3. Accompany the work with a written offer, valid for at least three years, to give the same user the materials specified in Subsection 6a, above, for a charge no more than the cost of performing this distribution.
   4. If distribution of the work is made by offering access to copy from a designated place, offer equivalent access to copy the above specified materials from the same place.
   5. Verify that the user has already received a copy of these materials or that you have already sent this user a copy.

   For an executable, the required form of the "work that uses the Library" must include any data and utility programs needed for reproducing the executable from it. However, as a special exception, the materials to be distributed need not include anything that is normally distributed (in either source or binary form) with the major components (compiler, kernel, and so on) of the operating system on which the executable runs, unless that component itself accompanies the executable.

   It may happen that this requirement contradicts the license restrictions of other proprietary libraries that do not normally accompany the operating system. Such a contradiction means you cannot use both them and the Library together in an executable that you distribute.
7. You may place library facilities that are a work based on the Library side-by-side in a single library together with other library facilities not covered by this License, and distribute such a combined library, provided that the separate distribution of the work based on the Library and of the other library facilities is otherwise permitted, and provided that you do these two things:

   1. Accompany the combined library with a copy of the same work based on the Library, uncombined with any other library facilities. This must be distributed under the terms of the Sections above.
   2. Give prominent notice with the combined library of the fact that part of it is a work based on the Library, and explaining where to find the accompanying uncombined form of the same work.
8. You may not copy, modify, sublicense, link with, or distribute the Library except as expressly provided under this License. Any attempt otherwise to copy, modify, sublicense, link with, or distribute the Library is void, and will automatically terminate your rights under this License. However, parties who have received copies, or rights, from you under this License will not have their licenses terminated so long as such parties remain in full compliance.
9. You are not required to accept this License, since you have not signed it. However, nothing else grants you permission to modify or distribute the Library or its derivative works. These actions are prohibited by law if you do not accept this License. Therefore, by modifying or distributing the Library (or any work based on the Library), you indicate your acceptance of this License to do so, and all its terms and conditions for copying, distributing or modifying the Library or works based on it.
10. Each time you redistribute the Library (or any work based on the Library), the recipient automatically receives a license from the original licensor to copy, distribute, link with or modify the Library subject to these terms and conditions. You may not impose any further restrictions on the recipients' exercise of the rights granted herein. You are not responsible for enforcing compliance by third parties with this License.
11. If, as a consequence of a court judgment or allegation of patent infringement or for any other reason (not limited to patent issues), conditions are imposed on you (whether by court order, agreement or otherwise) that contradict the conditions of this License, they do not excuse you from the conditions of this License. If you cannot distribute so as to satisfy simultaneously your obligations under this License and any other pertinent obligations, then as a consequence you may not distribute the Library at all. For example, if a patent license would not permit royalty-free redistribution of the Library by all those who receive copies directly or indirectly through you, then the only way you could satisfy both it and this License would be to refrain entirely from distribution of the Library.

    If any portion of this section is held invalid or unenforceable under any particular circumstance, the balance of the section is intended to apply, and the section as a whole is intended to apply in other circumstances.

    It is not the purpose of this section to induce you to infringe any patents or other property right claims or to contest validity of any such claims; this section has the sole purpose of protecting the integrity of the free software distribution system which is implemented by public license practices. Many people have made generous contributions to the wide range of software distributed through that system in reliance on consistent application of that system; it is up to the author/donor to decide if he or she is willing to distribute software through any other system and a licensee cannot impose that choice.

    This section is intended to make thoroughly clear what is believed to be a consequence of the rest of this License.
12. If the distribution and/or use of the Library is restricted in certain countries either by patents or by copyrighted interfaces, the original copyright holder who places the Library under this License may add an explicit geographical distribution limitation excluding those countries, so that distribution is permitted only in or among countries not thus excluded. In such case, this License incorporates the limitation as if written in the body of this License.
13. The Free Software Foundation may publish revised and/or new versions of the Lesser General Public License from time to time. Such new versions will be similar in spirit to the present version, but may differ in detail to address new problems or concerns.

    Each version is given a distinguishing version number. If the Library specifies a version number of this License which applies to it and "any later version", you have the option of following the terms and conditions either of that version or of any later version published by the Free Software Foundation. If the Library does not specify a license version number, you may choose any version ever published by the Free Software Foundation.
14. If you wish to incorporate parts of the Library into other free programs whose distribution conditions are incompatible with these, write to the author to ask for permission. For software which is copyrighted by the Free Software Foundation, write to the Free Software Foundation; we sometimes make exceptions for this. Our decision will be guided by the two goals of preserving the free status of all derivatives of our free software and of promoting the sharing and reuse of software generally.

No Warranty

15. Because the library is licensed free of charge, there is no warranty for the library, to the extent permitted by applicable law. Except when otherwise stated in writing the copyright holders and/or other parties provide the library "as is" without warranty of any kind, either expressed or implied, including, but not limited to, the implied warranties of merchantability and fitness for a particular purpose. The entire risk as to the quality and performance of the library is with you. Should the library prove defective, you assume the cost of all necessary servicing, repair or correction.
16. In no event unless required by applicable law or agreed to in writing will any copyright holder, or any other party who may modify and/or redistribute the library as permitted above, be liable to you for damages, including any general, special, incidental or consequential damages arising out of the use or inability to use the library (including but not limited to loss of data or data being rendered inaccurate or losses sustained by you or third parties or a failure of the library to operate with any other software), even if such holder or other party has been advised of the possibility of such damages.

Menu

About

Preview

Downloads

License

Search
